# Geographic Disparity of Severe Vision Loss — United States, 2009–2013

**Published:** 2015-05-22

**Authors:** Karen A. Kirtland, Jinan B. Saaddine, Linda S. Geiss, Ted J. Thompson, Mary F. Cotch, Paul P. Lee

**Affiliations:** 1 Division of Diabetes Translation, National Center for Chronic Disease Prevention and Health Promotion, CDC; 2 National Eye Institute, National Institutes of Health; 3 Department of Ophthalmology and Visual Sciences, University of Michigan Medical School

Vision loss and blindness are among the top 10 disabilities in the United States, causing substantial social, economic, and psychological effects, including increased morbidity, increased mortality, and decreased quality of life.* There are disparities in vision loss based on age, sex, race/ethnicity, socioeconomic status, and geographic location (1). Current surveillance activities using national and state surveys have characterized vision loss at national and state levels. However, there are limited data and research at local levels, where interventions and policy decisions to reduce the burden of vision loss and eliminate disparities are often developed and implemented. CDC analyzed data from the American Community Survey (ACS) to estimate county-level prevalence of severe vision loss (SVL) (being blind or having serious difficulty seeing even when wearing glasses) in the United States and to describe its geographic pattern and its association with poverty level. Distinct geographic patterns of SVL prevalence were found in the United States; 77.3% of counties in the top SVL prevalence quartile (≥4.2%) were located in the South. SVL was significantly correlated with poverty (r = 0.5); 437 counties were in the top quartiles for both SVL and poverty, and 83.1% of those counties were located in southern states. A better understanding of the underlying barriers and facilitators of access and use of eye care services at the local level is needed to enable the development of more effective interventions and policies, and to help planners and practitioners serve the growing population with and at risk for vision loss more efficiently.

Data from the 2009–2013 ACS were used to obtain county-level estimates of SVL and poverty level among adults aged ≥18 years and to examine the geographic pattern of SVL and its association with poverty. ACS, conducted by the U.S. Census Bureau, is an ongoing survey sent to approximately 250,000 addresses monthly, providing annual and multiyear estimates of demographic, housing, social and economic characteristics to communities.† In the 2009 ACS, the response rate for all states was 98.0%; in 2013, the response rate for all states was 89.9%.§ In 2008, ACS added questions about disability to better understand the population with disabilities, monitor against discrimination, distribute funds, and develop programs for persons with disabilities. ACS measures SVL based on responses to the question, “Is this person blind or does s/he have serious difficulty seeing even when wearing glasses.” In addition, ACS provides county-level data on the percentage of the residents living below the federal poverty level during the preceding 12 months. These estimates are based on reported family income and poverty thresholds published by the U.S. Census Bureau.

The ACS county-level estimates of SVL and poverty level from the combined 2009–2013 surveys were used in this analysis.¶ The 3,143 counties were divided into quartiles based on SVL prevalence. Counties were divided into quartiles based on the proportion of residents living below the poverty level. Prevalence of SVL and poverty were compared by U.S. Census regions. Pearson’s correlation was used to estimate the county-level association between SVL and poverty.

The prevalence of SVL among adults aged ≥18 years ranged from <1% to 18.4%, with a median of 3.1%. More than three quarters (77.3%) of the U.S. counties in the top quartile of SVL prevalence were located in the South, followed by 11.7% in the West, 10.7% in the Midwest, and 0.3% in the Northeast (Figure 1). The proportion of persons living below poverty level ranged from 1.3% to 48.8% across all counties, with a median of 13.9%. Nearly three quarters (74.5%) of these counties were located in the South, followed by 13.1% in the Midwest, 11.5% in the West, and 0.9% in the Northeast (Figure 2). Among counties in the top quartile for SVL prevalence, 55.5% were also in the top quartile of poverty, and county prevalence of SVL was significantly correlated with county poverty (r = 0.5, p<0.0001). Of the 437 counties in the top quartiles for both SVL and poverty, 83.1% were in the South, followed by 9.1% in the West and 7.8% in the Midwest (Figure 3). No county in the top quartile for both SVL prevalence and poverty were in the Northeast. Eight states had at least 6% of their counties in the top quartile for both SVL and poverty: Alabama, Arkansas, Georgia, Kentucky, Mississippi, North Carolina, Tennessee, and Texas.

## Discussion

Vision loss often affects activities of daily living, leads to depression and social isolation, and increases the risk for falls and injuries. An estimated 4 million persons aged ≥40 years in the United States are either blind or have vision loss (defined as best corrected visual acuity <20/40 in the better-seeing eye) and this number is projected to increase to 10 million by 2050.** The most common causes of vision loss among adults in order of prevalence are cataracts, diabetic retinopathy, glaucoma, and age-related macular degeneration, and all these causes of vision loss can develop gradually without warning signs.†† Many others have uncorrected refractive errors that could be easily corrected with glasses or contact lenses (2). According to the National Eye Institute, a comprehensive dilated eye examination by an eye-care professional can detect certain eye diseases and conditions in their early stages, before vision loss occurs, and identify uncorrected refractive errors (3). In addition to eye examinations, minimizing risks (e.g., by eating right, maintaining a healthy weight, not smoking, understanding one’s family eye health history, and using proper eye-safety practices) can prevent or delay vision impairment.§§ In 2013, the total cost of vision problems in the United States was estimated at $145 billion (2). Interventions to detect and manage eye conditions that can lead to vision loss are available and cost-effective (4). However, access and use of eye care services are often suboptimal (4), possibly because this is a preventive service that is frequently not covered by insurance carriers. Medicare, which serves the population at greatest risk for vision loss, persons aged ≥65 years, provides a benefit for comprehensive dilated eye examination only for persons with diabetes or at high risk for glaucoma (5).

This report describes the geographic distribution of SVL across all U.S. counties. Using ACS 5-year county estimates, this analysis found 2.6% of adults aged ≥18 years reported SVL. Prevalence varied by county, ranging from <1% to 18.4%, and counties with high SVL were concentrated in southern states. The data show the county level correlation of SVL and poverty, indicating that counties with higher levels of poverty had higher levels of SVL. These findings are consistent with U.S. Census reports of lower earnings and higher poverty rates among persons with disabilities.¶¶

A report on vision loss and work disability from the Institute of Medicine suggests that SVL can result in lower economic earnings (6). Previous studies on the link between a person’s lower socioeconomic status and visual impairment, eye diseases, and ocular risk factors have found that access to and use of health care are important factors in the relationship between visual impairment and socioeconomic status, and that persons with higher income and education were more likely than those with lower income to visit an eye care provider (4,7). One report demonstrated the association between low rates of eye care use and lack of health insurance coverage, in addition to limited coverage for ocular care in private insurance plans (8). In addition, international studies suggest that neighborhood environment impacts health outcomes through various means, such as unhealthy behaviors in the local environment, availability of health care services, lack of eye health knowledge, and in particular, lack of access to eye care (9,10).

The findings in this report are subject to at least three limitations. First, data regarding SVL are self-reported and subject to recall bias or social desirability bias, which might cause persons to claim that their vision is better than it is. Second, data are cross-sectional and do not allow conclusions on causality or biologic plausibility. However, these results are consistent with previous studies finding a correlation between poverty and vision loss. Vision loss could be a consequence of poverty (e.g., lack of access to care and lower use of preventive services). Alternatively, persons with SVL often have limited access to education and employment opportunities, which might in turn decrease their earning potential. Finally, the data represent crude estimates of SVL prevalence and the correlation between SVL and poverty, without accounting for other individual and county-level characteristics, such as race/ethnicity, age, and prevalence of related diseases.

The results of this study suggest geographic disparity in SVL, with the South disproportionately bearing a higher prevalence of SVL and poverty. Counties with high prevalence of SVL tended to be those with high poverty levels. Further investigations are needed to better understand the socio-demographic disparities of vision loss, how to minimize risk factors associated with vision loss, and how to improve access and use of eye care services. To reduce risks for vision loss, counties with high rates of SVL can promote healthy eating, maintaining a healthy weight, not smoking, understanding one’s family eye health history, using proper eye-safety practices, and routine eye examinations.

What is already known on this topic?

Vision loss often affects activities of daily living, leads to depression and social isolation, and increases the risk for falls and injuries. The U.S. government, in conjunction with the vision health communities, identified access to eye care services and the reduction of disparities in vision loss and as public health priorities in the *Healthy People 2020* national health objectives. There are limited vision loss data and research at local levels.

What is added by this report?

Distinct geographic patterns of severe vision loss prevalence were found at the county-level in the United States. The findings indicate a significant correlation between severe vision loss and poverty for U.S. counties. The highest prevalence of severe vision loss and poverty were found in the southern states.

**What are the implications for public health practice?**

To reduce risks for vision loss, counties with high rates of severe vision loss can promote healthy eating, maintaining a healthy weight, not smoking, understanding one’s family eye health history, using proper eye-safety practices, and routine eye examinations.

## Figures and Tables

**FIGURE 1:**
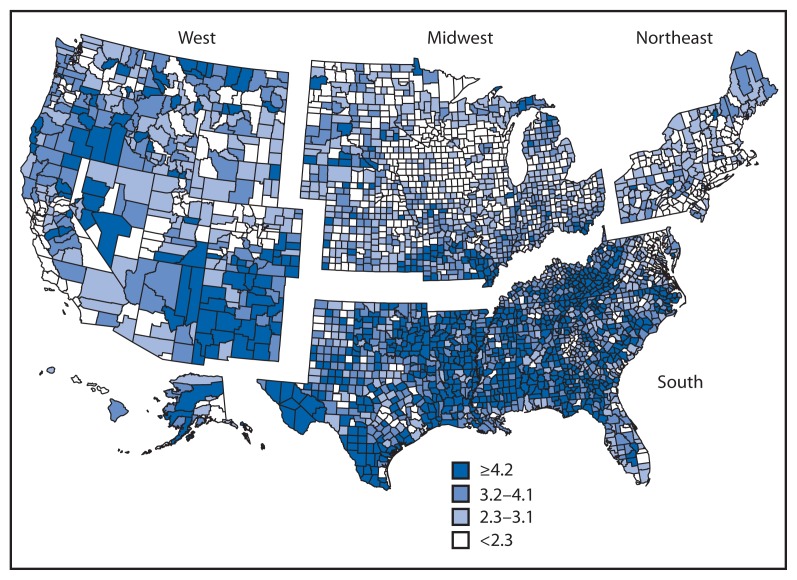
Percentages of persons aged ≥18 years with severe vision loss, by county* — United States, American Community Survey, 2009–2013 * Northeast = 217; Midwest = 1,055; South = 1,423; West = 448.

**FIGURE 2:**
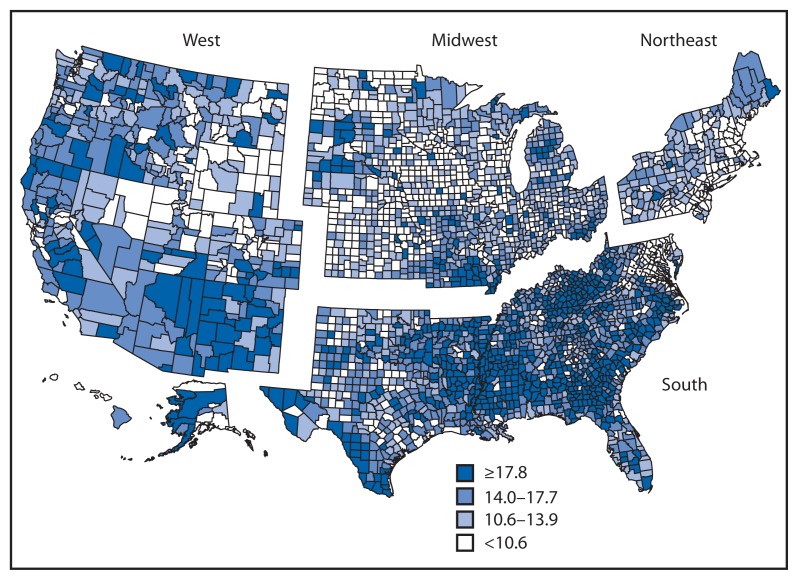
Percentages of persons aged ≥18 years with family income below poverty level, by county* — United States, American Community Survey, 2009–2013 * Northeast = 217; Midwest = 1,055; South = 1,423; West = 448.

**FIGURE 3:**
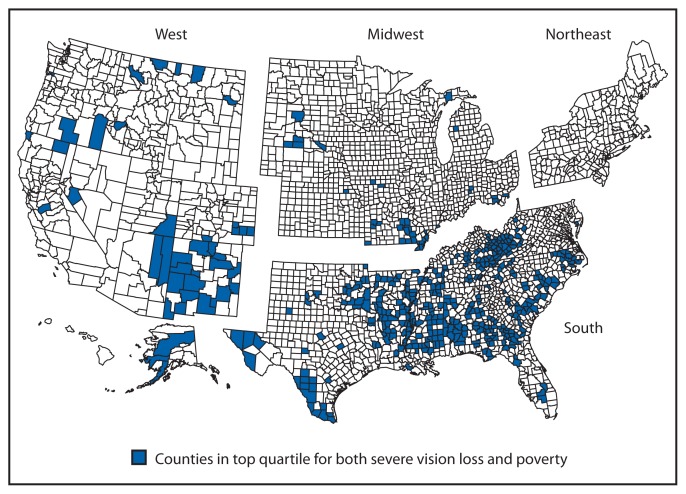
Counties* in the top quartile for both severe vision loss and family income below poverty level — United States, American Community Survey, 2009–2013 * Northeast = 217; Midwest = 1,055; South = 1,423; West = 448. Numbers indicate total number of counties for each U.S. Census region.

## Footnotes

* Information available at http://www.cdc.gov/visionhealth/pdf/improving_nations_vision_health.pdf.

† Information available at http://www.census.gov/acs/www/Downloads/data_documentation/SubjectDefinitions/2013_ACSSubjectDefinitions.pdf.

§ Information available at http://www.census.gov/acs/www/methodology/response_rates_data.

¶ Information available at http://dataferrett.census.gov/TheDataWeb/index.html.

** Information available at http://forecasting.preventblindness.org.

†† Information available at http://www.cdc.gov/visionhealth/basic_information/eye_disorders.htm.

§§ Information available at http://www.cdc.gov/visionhealth/healthyvisionmonth/index.htm.

¶¶ Information available at http://www.census.gov/prod/2012pubs/p70-131.pdf.
